# Achieving the fast track 90-90-90 and 95-95-95 targets in sub-Saharan Africa: A rapid review

**DOI:** 10.4102/jphia.v16i1.691

**Published:** 2025-04-21

**Authors:** Celenkosini T. Nxumalo, Usangiphile Buthelezi, Hlolisile Chiya, Mokgadi U. Makgobole, Nomakhosi Mpofana, Themba Mgwaba, Zamasomi Luvuno

**Affiliations:** 1 Department of Research Development and Postgraduate Support, Office of the DVC Research and Innovation, University of the Western Cape, Cape Town, South Africa; 2 Centre for Rural Health, School of Nursing and Public Health, University of KwaZulu-Natal, Durban, South Africa; 3 Discipline of Nursing, School of Nursing and Public Health, University of KwaZulu-Natal, Durban, South Africa; 4 Department of Somatology, Faculty of Health Sciences, Durban University of Technology, Durban, South Africa; 5 School of Built Environment, College of Humanities, University of KwaZulu-Natal, Durban, South Africa

**Keywords:** HIV, 90-90-90, HIV treatment cascade, 95-95-95, UNAIDS fast-track targets, best practices, recommendations, successes, challenges

## Abstract

**Background:**

The Joint United Nations Programme on HIV/AIDS (UNAIDS) set targets for 95% of people living with human immunodeficiency virus (HIV) infection to know their status, 95% to receive antiretroviral therapy (ART) and 95% to achieve viral suppression. These targets mirror the 90-90-90 targets that were aimed to be met by 2020 to end AIDS as a public health threat by 2030.

**Aim:**

The study aims to synthesise evidence on recommendations, best practices and challenges in achieving 90-90-90 escalated to 95-95-95 fast-track targets in sub-Saharan Africa.

**Setting:**

The review included empirical evidence from sub-Saharan Africa.

**Method:**

We followed Arksey and O’Malley’s methodological framework for this scoping review. A systematic search of relevant articles was conducted using electronic databases such as Scopus, EBSCOHost, PubMed, Science Direct and Sabinet. The results were reported using the Preferred Reporting Items for Systematic Reviews and Meta-Analyses (PRISMA) flow diagram.

**Results:**

There were 6943 relevant study titles that were identified from the five databases. Following duplicates removal, title and abstract screening, 31 articles were included for full-text review. A combination of qualitative, quantitative and mixed methods studies were included.

**Conclusion:**

The 90-90-90 targets have been achieved in part, but challenges remain, particularly for key and vulnerable populations. Even as successes and challenges towards ‘90-90-90 by 2020’ are outlined, new fast-track ‘95-95-95 by 2030’ targets have been established because of concerns that the original targets may not have achieved epidemic control.

**Contribution:**

The findings of this review have implications for policy and practice related to interventions to facilitate the realisation of HIV epidemic control as outlined by the UNAIDS 95-95-95 treatment cascade.

## Introduction

The fast-track treatment targets set by the Joint United Nations Programme on HIV/AIDS (UNAIDS) called for 90% of people living with human immunodeficiency virus (HIV) infection to know their status, 90% to receive antiretroviral therapy (ART) and 90% to achieve viral suppression by 2020.^1^ It is estimated that by achieving all three targets, 73% of people living with HIV globally will become virologically suppressed. An undetectable viral load is associated with a significant decrease in HIV-related morbidity and mortality, reduced risk of sexual transmission and a decline in HIV incidence at the population level.^2^ As an epidemiological milestone, the 90-90-90 and 95-95-95 targets aim to reduce new infections and AIDS-related mortality thus contributing to ending HIV and/or acquired immunodeficiency syndrome (AIDS) as an epidemic by 2030.^1,3,4^

Globally, the treatment cascade percentage was 84-73-66 in 2020, with sub-Saharan Africa (SSA) reporting lower rates than other regions.^5,6^ The world’s proportion of people living with HIV who knew their HIV status was estimated to be 84% in 2020, with women of child-bearing age (15–49 years) being more likely to be aware of their status than men.^5,7^ In 2020 globally, there were 28.2 million people living with HIV who were accessing ART treatment (73%), including 85% of pregnant women who had access to ART to prevent vertical HIV transmission.^5,8^ There has been significant progress in scaling up ART in low- and middle-income regions such as SSA over the past decade, with 15 million people now receiving ART.^9^ However, nearly 11 million people living with HIV needed to achieve the third ‘90’ (viral suppression) in 2018.^10^ In 2020, only 66% of those who were on treatment had viral suppression.^5^ Furthermore, in low- and middle-income countries, there are regional differences that exist with regard to viral suppression, with Eastern and Southern countries having better viral suppression (50%) compared to West and Central African countries (25%).^10^

Management of the HIV epidemic continues to lag because 6.1 million people living with HIV remain unaware of their HIV status and only 66% of those who know their status and are on treatment are virally suppressed, compromising efforts to achieve the UNAIDS fast-track targets.^8^ Various strategies and programmes have been implemented in an attempt to achieve the ‘90’ of the 90-90-90 targets. These include: the test and treat strategy, self-testing, prevention of mother-to-child transmission (PMTCT), adherence clubs, fast-track cities, targeting geographic hot-spots, focusing on vulnerable and key populations, awareness and treatment campaigns, and other community-based interventions. For example, through the PMTCT programme, ART coverage for pregnant women in southern and eastern Africa increased from 47% in 2010 to 93% in 2017^7^; adherence clubs have also been used to provide adherence support and expedite ART refills to keep people on treatment (Nachega et al.)^10^; targeting vulnerable (adolescent girls and young women, migrant populations, truckers, prisoners, soldiers, internally displaced people, refugees, and orphans and vulnerable children) and key populations (men who have sex with men, sex workers and their clients, people who inject drugs and transgender people)^11^ and to Fast-Track cities, to achieve 2020 (90-90-90) and 2030 HIV targets (95-95-95).^4,12^

While sub-Saharan Africa and other low- to middle-income countries have made significant progress towards achieving the previous 90-90-90 targets, many countries in the region had yet to do so by 2020. This means that key ‘leakages’ along the cascade remain, and these may be potential barriers to meeting the new targets. These include the rate of linkage to care and viral suppression among those receiving treatment.^4^ This scoping review thus aims to synthesise existing evidence on recommendations, best practices and challenges in achieving the 90-90-90 and 95-95-95 fast-track targets. The findings of this review thus provide a baseline of consolidated empirical evidence to inform interventions that may be implemented to support the realisation of the new 95-95-95 targets in SSA and other low- to middle-income regions.

## Methods

This systematic scoping review reports on the recommendations and best practices for the implementation of the fast-track 90-90-90 and 95-95-95 HIV treatment cascade in sub-Saharan Africa. The methodological framework by Arksey and O’Malley was used to guide the execution of the review.^13^ The following steps were subsequently followed when the review was carried out: (1) identification of the review question (2) identification of relevant studies, (3) selection of relevant studies, (4) charting of the data and (5) collating, summarising and reporting the findings. Quality appraisal of research studies was not done as the prime focus of this review was the relevance of studies in relation to the research question.

### Identifying the research question

The main research question is ‘*What are the recommendations, best practices, and challenges for implementation of the fast-track 90-90-90 and 95-95-95 HIV treatment cascade in sub-Saharan Africa*?’

The research sub-questions are:

- What is the existing evidence on the recommendations for the implementation of the fast-track 90-90-90 and 95-95-95 HIV treatment cascade in sub-Saharan Africa?
- What is the range of literature on the reported best practices for facilitating the implementation of the fast-track 90-90-90 and 95-95-95 HIV treatment cascade in sub-Saharan Africa?
- What are the successes and challenges that are reported in the literature to reach the fast-track 90-90-90 and 95-95-95 HIV treatment cascade targets in sub-Saharan Africa?

This study used the Population, Concept and Context (PCC) framework to align the study selection with the research question (Table 1).

**TABLE 1:** Population, concept and context framework.

Population	Concept	Context
People living with HIV and/or AIDS	Implementation of the fast-track 90-90-90 and 95-95-95 treatment cascade for HIV and/or AIDS	Sub-Saharan Africa

HIV, human immunodeficiency viruses; AIDS, acquired immunodeficiency syndrome.

### Identification of relevant studies

A combination of relevant search terms with Boolean operators was used to search for published and unpublished literature on the following databases: EBSCOhost (MEDLINE), PubMed, Scopus, Sabinet and Science Direct. Relevant grey literature was also obtained through a targeted search of reports, publications and websites from government and international organisations (i.e., UNAIDS, WHO, USAID, PEPFAR). Dissertations and theses on relevant search engines such as ProQuest Dissertations and Theses Global were also searched. Conference abstracts and proceedings were also searched from Citation Index.

The comprehensive search strategy was co-developed by the authors, subject specialists and university librarian to ensure the correct use of indexing terminology and Medical Subject Headings (MeSH) terms. The Boolean search terms were first piloted in PubMed to determine the relevance of the search terms and the scope of literature covered. The following search terms were used: ‘HIV treatment’ OR ‘90-90-90’ OR ‘95-95-95’ OR ‘treatment cascade’ OR ‘Fast-Track’ AND (Africa) as shown in Table 2. A snowball sampling approach was used to retrieve references from articles that were included but not identified by the search. A hand search was also performed on websites such as the World Health Organization (WHO) to identify potentially relevant literature.

**TABLE 2:** Results of pilot search in PubMed.

Date of search	Electronic database	Keywords/MeSH terms	Number of retrieved studies
01/09/2023	Pubmed	‘HIV treatment’ OR ‘90-90-90’ OR ‘95-95-95’ OR ‘treatment cascade’ OR ‘Fast-Track’ AND (Africa)	1978

HIV, human immunodeficiency viruses; MeSH, medical subject headings.

### Selection of eligible studies

#### Inclusion criteria

- Studies presenting evidence on people living with HIV and/or AIDS.
- Studies presenting evidence on the 90-90-90 and 95-95-95 treatment cascade and HIV Fast-track strategy.
- Studies presenting evidence from countries in the sub-Saharan African region.
- Studies published from the year 2014 onwards.
- English published studies.

#### Exclusion criteria

- Grey literature and studies conducted prior to 2014.
- Commentaries, opinion articles and editorials.
- Studies on fast-track 90-90-90 and 95-95-95 from countries outside sub-Saharan Africa.

An independent reviewer conducted the literature search and uploaded all emerging articles on Endnote X20 software. A thorough title screening was conducted by two reviewers, and all studies that did not address our research question were excluded together with all duplicates. The final Endnote database was shared for abstract and full-text screening. Two independent reviewers screened abstracts and full-text from all relevant records based on the specified inclusion and exclusion criteria. All efforts were made to obtain full-text articles for the review by engaging with experts in the field, contacting relevant authors and conducting a comprehensive search using the web. The assistance of an additional reviewer was sought where there were discrepancies that could not be resolved by discussion and mutual agreement. The included articles were then stored on Google Drive for charting on an Excel spreadsheet.

**FIGURE 1:**
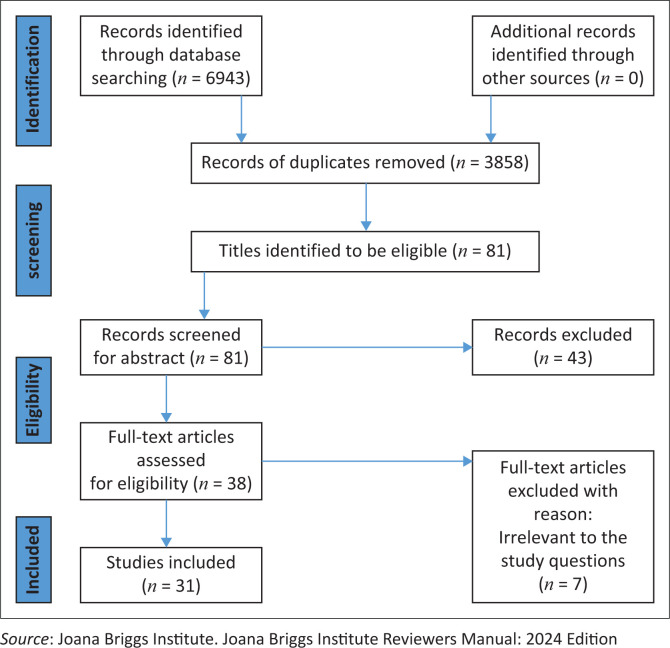
Preferred Reporting Items for Systematic Reviews and Meta-Analysis flowchart of the search process.

Using Cohen’s kappa statistic, the degree of agreement between the screeners’ results after screening abstracts and full articles was estimated. Values under 0.1 indicated no agreement, values between 0.10 and 0.20 indicated little to no agreement, values between 0.21 and 0.40 indicated fair agreements, 0.41–0.60 indicated moderate agreements, 0.61–80 indicated substantial agreements and values between 0.81 and 1.00 indicated almost perfect agreements. The study selection process will be summarised using the Preferred Reporting Items for Systematic Reviews and Meta-Analysis (PRISMA) diagram (Figure 1).^14^

### Charting the data

An electronic data charting template was developed to capture relevant information from the articles included in the review. The data charting form comprised elements from existing data charting tools but was designed by the authors to be unique for this review. The core components of our data extraction template contained the following fields: author, title, research objective, research methods, key findings and recommendations. This process ensured synthesis and interpretation of all studies that were identified in accordance to the inclusion and exclusion criteria of the review.

### Collating, summarising and reporting the findings

The findings of the review are presented narratively based on the following elements and outcomes: country of the study, current best practices and recommendations for implementation of the Fast-track 90-90-90 and 95-95-95 treatment cascade for HIV and/or AIDS in SSA.

## Results

There were 6943 relevant study titles that were identified from the five databases, and a total of 3858 duplicate studies that existed between databases were removed. Further exclusion of 3085 articles was done after title screening, and 43 studies were removed after the abstract review established that the content did not meet the inclusion criteria of this scoping review. Seven articles were excluded because of being irrelevant to the study research questions. Eventually, there were 31 full-text publications that were part of this scoping review’s final analysis (Figure 1). The studies included in this review comprised qualitative, quantitative and mixed-method studies. After an in-depth reading of selected studies, a thematic analysis of the selected studies was conducted. Research in SSA, the fast-track 90-90-90 and 95-95-95 treatment cascades addressed issues, best practices and suggestions.

The researches that were incorporated into this scoping review’s synthesis were from 14 different SSA countries. These included studies from South Africa (*n* = 7), Lesotho (*n* = 3), Malawi (*n* = 1), Tanzania (*n* = 2), Zimbabwe (*n* = 2), Nigeria (*n* = 1), Ethiopia (*n* = 5), Kenya (*n* = 1), Uganda (*n* = 3), South Sudan (*n* = 1), Côte d’Ivoire (*n* = 1), Zambia (*n* = 1), Mozambique (*n* = 1), Botswana (*n* = 1) and one study included 12 SSA countries.

## Discussion

This scoping review identified, described and synthesised the existing evidence on the recommendations, current best practices, successes and challenges for facilitating the implementation of the fast-track 90-90-90 and the new fast-tack 95-95-95 targets for HIV treatment cascade in sub-Saharan African countries.

In the first nationwide survey to gauge progress towards the UNAIDS 90-90-90 targets in 2016, Hakim et al.^15^ found a 14.1% HIV prevalence in Zimbabwe. Of those living with HIV, 76.8% (95% confidence interval [CI]: 74.9–78.7) were aware of their status or had detectable antiretroviral levels. Of these, 88.4% (95% CI: 87.1–89.7) were receiving ART, and of these people, 85.3% (95% CI: 83.4–87.1) had viral load suppression. Having at least one sexual partner and being 15–34 years of age were associated with not knowing that one was HIV-positive. Labhardt et al.^16^ carried out a second cohort study to look into the ‘last 90’ in Lesotho. They found that retention in care and viral suppression among patients with persistent low-grade viremia is linked to viral resuppression after enhanced adherence counselling (EAC) (aOR: 5.02; 95% CI: 1.14–22.09; *p* = 0.033) and switching to second-line after EAC when viremia persists (aOR: 7.17; 1.90–27.04; *p* = 0.004). Furthermore, ART treatment time, level of VL, age, gender and education were not associated.^16^

Tanzania was far from meeting the first ‘90’ of the 90-90-90 targets, according to a study by Wang et al.^17^ The three targets were assessed at 61–90–85 using the weighted analysis of the population-based HIV impact assessment survey from 2016 to 2017. The results of this study also indicated that eliminating the HIV epidemic by 2030 will require raising knowledge of HIV status.^17^ Therefore, these results suggest that prioritising interventions aimed at the first ‘90’ will help Tanzania contain the HIV pandemic.

In South Africa, the adoption of multifaceted quality improvement approaches such as the introduction of facility-based viral load champions has been shown to be effective in facilitating improvement of the last ‘90’.^18^ The findings of a study by Sunpath et al.,^18^ showed that viral load completion rates at three different sites improved from 68% (*n* = 140/205 patients), 54% (*n* = 84/155 patients) and 64% (*n* = 323/504 patients), to a completion rate of 83% (*n* = 995/1194 patients), 90% (*n* = 793/878 patients) and 99% (*n* = 3101/3124 patients) (*p* = 0.0001) respectively, after the adoption of this quality improvement strategy. Additionally, according to Marinda et al.,^19^ in order for South Africa to meet the 90-90-90 targets by 2020, 6 720 589 people living with HIV who are 15 years of age or older must be diagnosed, 6 048 530 must begin ART, and 5 443 677 must be receiving ART in order to achieve viral suppression. Towards meeting the UNAIDS 90-90-90 targets, significant progress has been accomplished in the context of high HIV prevalence. According to a study carried out in Mbongolwane and Eshowe, KwaZulu-Natal, South Africa, both men and women on ART were able to suppress their viral infections. However, there are still gaps in HIV diagnosis and ART coverage, particularly for men and people under 30.^20^

Progress has also been made by other countries towards achieving not only the 90-90-90 targets but the 95-95-95 as well. In a research carried out in Botswana by Lebelonyane et al.,^21^ 93% (*n* = 13 328 of 14 270) of participants knew they had HIV, 93% (*n* = 12 259 of 13 124) were receiving ART, 98% (*n* = 11 687 of 11 954) had suppressed viral loads. Ethiopia was another nation that was demonstrated to be well on its way to reaching the 90-90-90 targets and lowering HIV-related fatalities. Human immunodeficiency virus-related mortality decreased by 58% between 2011 and 2016; however, the number of new HIV infections decreased by just 6% during the same period. In 2011, discriminatory views amounted to 77.9%; by 2016, they had dropped to 41.5%. In 2016, around 79% of persons living with HIV were aware of their status; of those who were, 90% were on ART, and 88% had viral suppression.^22^ Despite evidence to suggest that Ethiopia may be on track to achieving the fast-track targets, reduction of new rates of HIV infections, persistence of stigma and discrimination and decline in funding to support the HIV response strategy remain a challenge. Strategies towards eliminating HIV and/or AIDS should incorporate prevention and enhanced treatment approaches for key and vulnerable populations on the basis of geographical location.

Lastly, accelerating the response to meet the UNAIDS target has been shown by different studies to prevent a significant number of new HIV infections, especially in the long term, and that viral suppression is the backbone of epidemic control and of reaching the 95/95/95 target.^23,24^

### Successes and challenges

There were various successes that were identified by this scoping review in different African countries which included condom use during sex work as a highly effective strategy to prevent HIV transmission in the last decades in Côte d’Ivoire,^23^ use of quality improvement approach to managing poor viral suppression^25^ and the success of Mother-to-Child Transmission (PMTCT) programmes in linking a high proportion of diagnosed pregnant women to care and preventing vertical HIV transmission.^19^ Other studies showed a high proportion of the population achieving viral suppression in resource-limited settings.^26^ For example, Botswana was able to reach its 95-95-95 targets by testing men and youth for HIV.^21^ Furthermore, with universal testing, linkage interventions and ART in several nations, knowledge of HIV status and ART coverage grew towards 95–95–95 targets. Private–public collaboration was another strategy that was shown to be successful.^27^ A study by Onyango et al.^27^ carried out in Ethiopia showed that more people who are not yet aware of their HIV status can be reached and linked to care when HIV testing is strengthened in large hospitals and private, not-for-profit facilities and targeted outreach is increased to hard-to-reach or underserved populations. Ethiopia is also among the nations that was on the verge of accomplishing its 2020 goal of 90-90-90. The findings of an Ethiopia population-based HIV impact assessment survey revealed 97.1% (95% CI: 95.0–98.3) were on ART, of which 87.6% (95% CI: 83.9–90.5) achieved viral load suppression.^28^

Despite the aforementioned successes, there were also challenges that existed when it came to reaching the UNAIDS targets in African countries. These included issues with data management consistency and accuracy to track the UNAIDS targets.^29^ Moreover, the omission of childhood tuberculosis (TB)/HIV from global plans and reporting mechanisms contributes to challenges in terms of attaining an accurate and comprehensive understanding on the state of the 90-90-90 and 95-95-95 treatment cascade. There was a high prevalence of HIV-positive ignorance among men and adults under 35 years of age (this is especially true for male adolescents compared to their female counterparts and for those with little to no education).^15,30^ Additionally, geographical distance to access services,^31^ gender-based violence (leading to the limited-negotiating ability for safer sex), all contribute to increasing incidence and prevalence of HIV and/or AIDS thereby hindering efforts made towards realisation of the fast-track targets. Data gaps because of incomplete medical records leading to delayed ART initiation, and sex work being criminalised in other countries, and therefore, resulting in the implementation of laws prohibiting sex work are linked to a reduced capacity to negotiate safer sexual practices and a higher risk of HIV transmission.^32^ In addition, age, gender, rural–urban residence, education and wealth can be observed as socioeconomic inequalities that can hinder achieving the UNAIDS targets.^33^

### Best practices as shown by different studies across sub-Saharan Africa

- Lesotho’s successful scale-up of ART was largely because of task-shifting and decentralisation of ART provision to all nurse-led clinics.^16^
- By focusing a multifaceted quality improvement strategy using a clinic-level viral load champion and scaling up the intervention, the third 90 of the UNAIDS 90-90-90 target can be accomplished.^18^
- Identifying types of health facilities where people with undiagnosed HIV infection are more likely to be treated can improve HIV testing efficiency.^27^
- When HIV testing services are offered in big hospitals and non-profit organisations, together with focused outreach to underserved and difficult-to-reach populations, more people living with HIV and/or AIDS can be reached and referred to care.^27^
- Human immunodeficiency virus positivity and referral rates are high when partners are notified. For this reason, interventions that involve partners are useful for linking patients to care.^31^
- For 90–90–90 targets to be achieved, we must combine intensified interventions promoting prevention and treatment.
- Achieving the UNAIDS targets depends on a number of factors, including provider-initiated HIV testing and counselling (PITC), ART, treatment for opportunistic infections (OIs), voluntary counselling and testing (VCT) and PMTCT.^34^ The elimination of social and structural barriers that hinder access to comprehensive HIV prevention and treatment services remains a crucial strategy particularly among key populations. Human immunodeficiency virus testing services that promote retention and linkage into care through differentiated treatment provision models have also been found to be instrumental towards achieving and maintaining progress towards fast-track targets.
- Lay counsellors’ patient support initiatives have shown to improve programme and patient outcomes, and they will be essential to meeting the 90-90-90 and 95-95-95 targets, as well as the HIV targets in national strategic plans.^35^

### Recommendations

Programmes must prioritise prompt EAC and transition to second-line treatment for individuals whose viremia persists despite EAC in order to improve outcomes for patients who fail the last ‘90’.^16^ However, despite regular medicine availability, counselling training and flexible and frequent clinic days, a study by Ndikabona et al.^36^ on intense adherence counselling revealed that the viral suppression rate following intensive adherence counselling (IAC) failed to meet suggested targets. As a result, with people living with HIV, the IAC might not be sufficient to achieve viral suppression on its own. Other supplementary services should be used in conjunction with IAC to increase viral suppression rates following IAC. Conversely, the findings of this study also showed that patients with a higher viral load at the start of IAC and having a history of viral rebound was associated with a reduced likelihood of attaining viral suppression. The findings of other similar studies have also shown that the availability of different regimens free-of-charge may be associated with viral suppression. Moreover, timely viral load monitoring and use of genotypic resistance testing to guide treatment changes are other supplemental services that should be used with IAC to ensure attainment of fast-track targets.

In order to optimise community benefits and minimise stigma and marginalisation, testing is essential in vulnerable communities.^17^ In order to achieve the fast-track targets, low- and middle-income countries need integrated strategies.^17^ In a study conducted in Zambia by Sikazwe et al.,^29^ compared to patients who remained in their original facility, patients who were lost from one facility and reported participation in a new facility had a much higher rate of viremia: Despite the relatively low frequency of durable discontinuance from care, therefore, strategies are needed to consistently engage patients to enhance retention.

It is imperative that government HIV programmes collaborate with private, non-profit clinics. It has been demonstrated that this partnership model can significantly lower HIV incidence and mortality by increasing the yield of HIV tests, identifying People Living with HIV and/or AIDS (PLWHA) in high-yield subpopulations, and perhaps enhancing the accessibility of care for those who have not yet received a diagnosis.^27^ Better supply chain management can reduce drug stock-outs, novel treatments may be created to boost viral suppression and supported models of care can all be leveraged to promote viral suppression.^37^

It is crucial to gather data at the district and sub-district levels in order to undertake focused interventions at these levels and provide more actionable, trackable recommendations and results.^19^ Additionally, targeted interventions designed for particular provinces and regions are required.^19^ Other issues such substance misuse, poor health, mental health and other social and/or welfare aspects need to be addressed in order to ensure effective retention in care and adherence to treatment.^19^ Carrasco et al.^38^ state that as there is a strong link between men’s ignorance of HIV prevention, acceptance of gender based violence (GBV), discriminatory attitudes and never having been tested for the virus, it is critical to concentrate on HIV prevention, GBV prevention, and stigma reduction programmes aimed at men. It is necessary to use specialised approaches to reach guys.^38^

To increase awareness, treatment and viral load suppression, the national HIV care and treatment programme must focus on demographic categories with limited access to HIV services, such as men, young people and the uneducated.^28^ To comprehend the role of the individual, community and structural level hurdles to achieving the updated interim 95-95-95, more exploratory study is required.^28^

Although it has been underutilised in SSA,^31^ the acceptability, feasibility and efficacy of a partner notification and referral strategy to HIV/AIDS testing services (HTS) have been shown to be extremely beneficial in detecting individuals with undetected HIV infection.^9,10,11^ In order to improve the HIV treatment and care cascade in South Africa, more focus should be placed on promoting and raising demand for the various HIV testing models as well as enhancing formal and HIV-related education. This will help to reduce disparities in HIV testing and awareness within the nation.^30^

Compared to their less educated counterparts, the educated may have been exposed to more information about HIV, be more aware of the benefits of testing for the virus and be able to make an informed decision to get tested for it. Equity is a crucial component of closing this gap, and it can so be done by using home, mobile and community-based tactics in conjunction with community engagement to encourage HIV testing uptake.^30^ Promoting HIV testing at community gatherings for those without formal education is one tactic that can be utilised to close this gap and meet the first ‘90 target.^30^

To increase access and utilisation and meet the UNAIDS target, programmes must address issues such as HIV stigma, shortages of drugs and staff and long travel times to medical facilities.^39^ Governments ought to increase fair 95−95−95 target actions, giving the reduction of wealth-related disparities, age, rural-urban and educational disparities top priority.^33^ Lastly, combating the HIV/AIDS epidemic calls for a coordinated effort that includes treatment and prevention geared toward specific groups and areas as well as more financing.^22^

## Conclusion

While some of the 90-90-90 targets have been met, there are still numerous obstacles to overcome, particularly for vulnerable and important populations. New fast-track ‘95-95-95 by 2030’ targets have been set because of worries that the old targets may not have achieved epidemic control, even as successes and obstacles toward ‘90-90-90 by 2020’ are detailed. However, it is critical to expand evidence-based interventions that are affordable, accessible, community-based and well-received by individuals living with HIV. Furthermore, meeting the 90-90-90 and 95-95-95 targets will necessitate a continued financial and political commitment in addition to the quick adoption of certain methods for systematic improvement.^3^

Because they offer consolidated evidence that influences policy and practice in African nations, particularly in the SSA region, the review’s conclusions may have an impact on research, health policy and clinical practice. The findings also help facilitate reaching the fast-track target of UNAIDS 2030 to end the HIV and/or AIDS epidemic, through identifying best practices and implementation strategies that have worked in different countries across Africa. A limitation of this review is that the data that were included were limited to SSA countries and therefore may not have direct implications for high-income regions. Nonetheless, the findings may provide valuable insights for interventions that may be implemented in other low- to middle-income contexts.
